# Photo-induced-photo-catalytic SERS with silver-deposited TiO_2_ nanorods for ultrasensitive and sustainable detection of low Raman cross-section molecules

**DOI:** 10.1039/d5ra01238d

**Published:** 2025-04-24

**Authors:** Quan-Doan Mai, Dang Thi Hanh Trang, Ngo Thi Loan, Nhu Hoa Tran Thi, Ong Van Hoang, Ta Ngoc Bach, Nguyen Quang Hoa, Anh-Tuan Pham, Anh-Tuan Le

**Affiliations:** a Phenikaa University Nano Institute (PHENA), Phenikaa University Hanoi 12116 Vietnam doan.maiquan@phenikaa-uni.edu.vn tuan.leanh@phenikaa-uni.edu.vn; b Faculty of Materials Science and Technology, University of Science Ho Chi Minh City Vietnam; c University of Transport Technology Trieu Khuc, Thanh Xuan District Hanoi Vietnam; d Institute of Materials Science (IMS), Vietnam Academy of Science and Technology 18 Hoang Quoc Viet Hanoi 10000 Vietnam; e Faculty of Physics, VNU University of Science, Vietnam National University, Hanoi Thanh Xuan Hanoi Vietnam; f Faculty of Biotechnology, Chemistry and Environmental Engineering Hanoi 12116 Vietnam

## Abstract

Surface-enhanced Raman spectroscopy (SERS) offers significant advantages, including label-free, non-invasive analysis and ultrasensitivity down to the single-molecule level, making it widely applicable in analytical chemistry and biology. However, its effectiveness is limited when detecting molecules with inherently low Raman scattering cross-sections, restricting its broader applications. In this study, we apply the photo-induced-photo-catalytic SERS (PI-PC SERS) technique, utilizing an Ag-deposited TiO_2_ nanorod (Ag/TiO_2_ NR) substrate to overcome this limitation. The PI-PC SERS technique combines two optoelectronic effects: photo-induced enhanced Raman scattering (PIERS) and the photocatalytic activity of the metal/semiconductor substrate. PIERS amplifies Raman signals beyond normal SERS, while the photocatalytic effect facilitates the removal of residual analytes. The PI-PC SERS process follows three sequential irradiation steps: (i) pre-irradiation with 365 nm UV light to activate PIERS, (ii) laser excitation at 785 nm to capture the enhanced Raman signal, and (iii) post-irradiation with 365 nm UV light to trigger photocatalytic degradation. Two low Raman cross-section molecules, 4-nitrophenol (a widely used pesticide) and urea (an important biomarker), were selected to evaluate the performance of the PI-PC SERS technique on the Ag/TiO_2_ NR substrate. The results demonstrated that PI-PC SERS not only enhanced detection sensitivity tenfold compared to normal SERS but also enabled self-cleaning by efficiently removing residual analytes after measurement, ensuring substrate reusability. These findings pave the way for advancing SERS-based techniques for detecting low Raman cross-section molecules while broadening their potential applications in chemical and biological sensing fields.

## Abstract 2

In this study, we introduce a PI-PC SERS strategy based on Ag-deposited TiO_2_ nanorods (Ag/TiO_2_ NRs), integrating PIERS and photocatalysis effects to achieve ultrasensitive, renewable detection of low-Raman-cross-section molecules.

## Graphical abstract

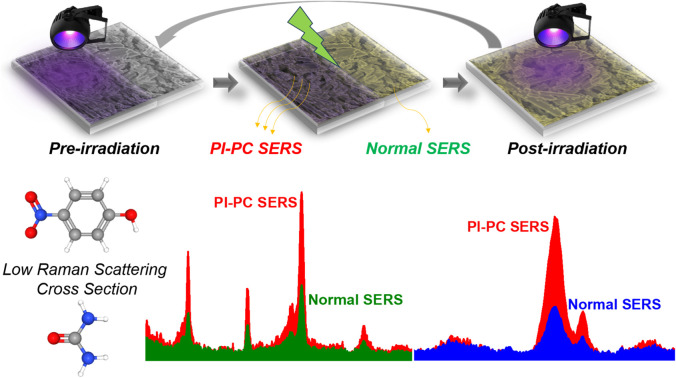

## 1. Introduction

Surface-enhanced Raman spectroscopy (SERS) sensors, known for their unique advantages such as ultrasensitivity down to the single-molecule level, label-free, and non-invasive detection, have attracted significant attention and found widespread applications in analytical chemistry (*e.g.*, food safety, water quality) and bioanalysis (*e.g.*, early diagnostics, pharmaceutical quality).^1–5^ The recent advances in nanoscience have further propelled SERS research, as purposefully designed nanostructures effectively exploit their unique properties to achieve significantly enhanced SERS signals while maintaining reliable performance.^4,6–8^ These nanostructures, ranging from simple colloidal and self-assembled metallic nanoparticles to more complex systems like nanocomposites, and even novel techniques based on the optical, electronic, and thermal properties of nanomaterials, have made notable strides in advancing SERS applications.^9–17^ Despite its remarkable sensitivity and widespread use, SERS faces an inherent limitation when detecting molecules with low Raman cross-sections. These molecules, due to their intrinsic low scattering efficiency, produce weak SERS signals, making detection challenging.^18,19^ The Raman cross-section, typically expressed in cm^2^ sr^−1^ per molecule, defines the probability of a photon being scattered inelastically by a given molecule.^20^ High-scattering analytes such as rhodamine 6G possess high Raman cross-sections on the order of 10^−25^ cm^2^ sr^−1^, yielding strong SERS signals that facilitate easy detection.^21,22^ Conversely, small or weakly polar molecules such as gases, urea, or 4-nitrophenol exhibit much lower Raman cross-sections (∼10^−30^ to 10^−27^ cm^2^ sr^−1^), often resulting in poor signal enhancement even under optimized SERS conditions.^23,24^ As a consequence, detection limits for these targets generally remain in the micromolar range (10^−3^ to 10^−4^ M), limiting their practical utility. Nonetheless, many low Raman cross-section molecules hold substantial value across various domains such as food safety and healthcare diagnostics. Thus, developing strategies to enhance SERS detection for these molecules could significantly expand the potential applications and impact of this powerful sensing platform.

Among the nanomaterials developed for SERS, metal/semiconductor structures have received significant attention due to the synergistic integration of two key enhancement mechanisms: electromagnetic (EM) and chemical (CM), resulting in significantly enhanced overall SERS performance.^25–27^ In addition, several intriguing physical and chemical effects in these structures have been leveraged to improve sensor efficacy, offering advantages such as enhanced stability and cost-effectiveness.^16,27,28^ In 2016, Ben-Jaber *et al.* discovered the photo-induced enhanced Raman scattering (PIERS) effect on SERS substrates based on metal/semiconductor structures by pre-irradiating SERS substrates composed of gold (Au) nanoparticles on a TiO_2_ surface, leading to a remarkable increase in sensing performance compared to conventional SERS for a wide range of molecular targets.^16^ Since then, this technique has been explored and applied to various metal/semiconductor-based SERS substrates and diverse target molecules, resulting in even further optimization of sensing performance.^29–31^ The enhancement effect of PIERS, as established in prior studies, may have the potential to improve the SERS signal of low Raman cross-section molecules – where normal SERS techniques often fall short. In 2022, by implementing PIERS on Ag/TiO_2_ nanoparticle substrates (with TiO_2_ in particle shape and anatase phase), we achieved significantly improved detection of urea and 4-nitrophenol as representative low Raman cross-section molecules.^32^ The system reached a detection limit as low as 10^−6^ M, representing an enhancement of two to three orders of magnitude over normal SERS. Beyond that, in light of growing real-world demands, there is increasing interest in the development of SERS substrates that not only provide high sensitivity but also offer additional features such as reusability, aiming to reduce operational costs and improve sustainability. Most recently, we introduced the photo-induced-photo-catalytic (PI-PC SERS) technique, which combines the PIERS effect with photocatalysis in metal/semiconductor nanostructures to achieve superior sensing performance and reusability compared to normal SERS.^33^ This technique has been successfully applied to Raman-sensitive molecules, such as methylene blue (a dye) and thiram (a pesticide). Applying the PI-PC SERS technique to low Raman cross-section molecules may offer not only the advantage of enhancing their weak SERS signals to improve sensitivity, but also the potential to meet other practical demands such as reducing operational costs and improving sustainability. Moreover, since the semiconductor component plays a central role in both PIERS and photocatalysis, variations in its properties – such as morphology and crystal phase – can significantly influence the overall performance of the PI-PC SERS technique.

In this study, we developed Ag-deposited TiO_2_ nanorods (Ag/TiO_2_ NRs – with TiO_2_ in rod shape and rutile phase) as metal (Ag)/semiconductor (TiO_2_) SERS substrates and applied the PI-PC SERS technique to improve the sensing performance for low Raman cross-section molecules, namely 4-nitrophenol (4-NP, a high-priority toxic pollutant and widely used pesticide, important in food safety) and urea (a biomarker for early diagnosis, relevant to kidney and cardiovascular diseases), compared to normal SERS. The results showed that the PI-PC SERS technique significantly outperforms normal SERS on the Ag/TiO_2_ NR substrate for both 4-nitrophenol and urea. Specifically, for 4-nitrophenol, PI-PC SERS achieved a detection limit of 6.8 × 10^−7^ M, approximately 10 times better than the case of normal SERS with a detection limit of 8.1 × 10^−6^ M. A similar result was observed for urea, with PI-PC SERS reaching a detection limit of 6.9 × 10^−7^ M, compared to only 8.9 × 10^−6^ M for normal SERS. Furthermore, the photocatalysis of the Ag/TiO_2_ NR substrate in PI-PC SERS was demonstrated, showing that after the sensing process, residual 4-NP and urea molecules on surface of the Ag/TiO_2_ NRs were efficiently removed *via* photocatalytic degradation, providing self-cleaning and reusability, thus ensuring cost-efficiency and sustainability. The enhanced sensing capability for low Raman cross-section molecules makes the PI-PC SERS technique a promising approach for applications in food safety, early diagnosis, and other fields involving low Raman cross-section molecules.

## 2. Materials and methods

### 2.1. Materials

The precursors, including silver nitrate (AgNO_3_, ≥99.0%), sodium borohydride (NaBH_4_, 99%), ethanol (C_2_H_5_OH, 98%), and cetyltrimethylammonium bromide (C_19_H_42_NBr, 99.9%), were procured from Shanghai Chemical Reagent and utilized without any additional purification. The low Raman cross-section molecules, namely 4-nitrophenol (C_6_H_5_NO_3_, ≥99%) and urea (CO(NH_2_)_2_, 99%), were obtained from Sigma-Aldrich. Titanium foils (99.99% purity) with dimensions of 100 mm × 20 mm × 1 mm were used in the experiments. All procedures were carried out using double-distilled water.

### 2.2. Synthesis of Ag-deposited TiO_2_ nanorods material and their characterizations

Ag-deposited TiO_2_ nanorods (Ag/TiO_2_ NRs) were fabricated using a two-step process. Initially, TiO_2_ nanorods were formed by an electrochemical method, followed by the deposition of Ag nanoparticles onto the TiO_2_ substrate through chemical reduction. The electrochemical fabrication process of TiO_2_ nanorods is described in detail in our previous study, with slight modifications in the applied voltage, reaction time, and calcination temperature, which may lead to differences in morphology and crystal phase composition.^34^ The reaction system consisted of three key components: two parallel titanium electrodes spaced 3 cm apart, a direct current (DC) power supply, and an electrolyte solution in a 200 mL beaker. The electrolyte solution consisted of 0.1 M cetyltrimethylammonium bromide (CTAB) dispersed in 200 mL of distilled water. The TiO_2_ nanorod formation reaction occurred over 5 hours under a 25 V DC current applied across the titanium electrodes. The resulting bright white solution containing the TiO_2_ nanorods was dried at 80 °C for 6 hours to obtain TiO_2_ powder. Subsequently, the TiO_2_ was annealed at 750 °C for 4 hours to achieve a crystalline structure. After the thermal treatment, the resulting crystalline TiO_2_ powder appeared bright white. The Ag/TiO_2_ NR material was formed through the reduction of AgNO_3_ in the presence of TiO_2_. First, 100 mg of crystalline TiO_2_ nanorods were then dispersed in 100 mL of distilled water under ultrasonic agitation for 15 minutes and then stirred uniformly. A calculated amount of 158 mg of AgNO_3_, dispersed in 20 mL of distilled water, was added and stirred for 1 hour to ensure optimal interaction between the Ag^+^ ions and TiO_2_. Afterward, a 10 mL solution containing 35 mg of NaBH_4_ was gradually introduced to reduce the Ag^+^ ions to Ag nanoparticles. During the addition of NaBH_4_, the solution color changed from bright white to brown, indicating the reduction of Ag^+^ ions into Ag nanomaterial. After the addition of NaBH_4_, the reaction was allowed to proceed for an additional hour to ensure complete reduction. The final resulting solution containing Ag/TiO_2_ NRs was used directly for subsequent experiments. The Ag to TiO_2_ ratio was selected as 1 : 1, as this ratio has been shown to provide optimal SERS enhancement in previous studies.^32^ The morphology and structure of the Ag/TiO_2_ NRs were analyzed using a field emission scanning electron microscopy (FE-SEM, Hitachi S-4800). The samples were prepared by drop-casting the Ag/TiO_2_ NR suspension onto a clean silicon wafer and drying under ambient conditions. Imaging was performed under high vacuum at an accelerating voltage of 5 kV, providing high-resolution surface morphology observations. Crystalline properties of Ag/TiO_2_ NRs were investigated by X-ray diffraction (XRD) analysis was performed using a Bruker D5005 diffractometer equipped with a Cu Kα radiation source (*λ* = 1.5406 Å). The measurements were conducted at an operating voltage of 40 kV and a current of 30 mA. Diffraction patterns were recorded over a 2*θ* range of 20° to 80°, with a step size of 0.05° and a counting time of 1 second per step. Prior to analysis, the sample were prepared by drop-casting the Ag/TiO_2_ NR suspension onto a clean silicon wafer, followed by drying under ambient conditions. The chemical properties and surface interactions of the Ag/TiO_2_ NRs were examined using Raman spectroscopy with a MacroRaman™ Raman spectrometer (Horiba) using a 785 nm laser for excitation. The spectra were collected in the range of 200 cm^−1^ to 2000 cm^−1^ with a spectral resolution of 1 cm^−1^. The laser power was set to 10 mW, and the exposure time was set to 30 seconds for signal acquisition.

### 2.3. Substrate preparation, SERS and PI-PC SERS measurements

The SERS substrate was prepared by depositing the material onto an aluminum (Al) base with dimensions of 1 cm × 1 cm × 0.1 cm, featuring a circular hole of 0.2 cm in diameter on the surface where the material was deposited. The Al base was first cleaned with ethanol and then allowed to dry naturally at room temperature (RT). A suspension of Ag/TiO_2_ NRs was drop-cast onto the active surface area and allowed to dry at room temperature. Aqueous solutions of 4-NP (ranging from 10^−3^ M to 5 × 10^−7^ M) and urea (10^−3^ M to 5 × 10^−7^ M) were prepared at different concentrations (the pH of the mixture was 7, as distilled water was used as the solvent and no pH-adjusting agents were added). For each SERS measurement, 5 μL of the analyte solution was deposited onto the prepared substrate and left to dry naturally at room temperature. Raman spectra were collected using a MacroRamamTM spectrometer (Horiba) with a 785 nm laser excitation source. Measurements were performed with a 100× objective lens (numerical aperture: 0.90), while the laser power was maintained at 45 mW at a 45° contact angle. The laser spot had a diffraction-limited diameter of 1.1 μm (calculated as 1.22*λ*/NA) with a focal depth of 115 nm. Each spectrum was obtained with an exposure time of 10 s and three accumulations, followed by baseline correction to ensure data accuracy.

The PI-PC SERS technique was performed as described in detail in our previous study.^33^ The PIERS effect of the Ag/TiO_2_ NR SERS substrate was activated by pre-irradiating the substrate with light at a wavelength of 365 nm for 30 minutes. The sensing signal was then collected immediately to ensure optimal retention of the PIERS effect. The self-cleaning capability of the Ag/TiO_2_ NR SERS substrate was demonstrated by activating the photocatalytic effect of the Ag/TiO_2_ NRs through post-irradiation of the SERS substrate containing residual analyte, using light at a wavelength of 365 nm. The degradation of the residual analyte molecules on the SERS substrate was monitored in real-time by observing the SERS spectrum and the intensity of their characteristic peaks. Finally, the SERS substrate was deemed reusable when the characteristic peaks of the residual analyte were no longer visible in the SERS spectrum.

### 2.4. Calculation of limit of detection (LOD)

The LOD value was calculated based on the linear equation established for each technique (SERS, PI-PC SERS) and the Raman signal of the corresponding analyte in its powder form. The LOD is calculated using the following equation:^35^

1 LOD = 10^[(*Y*_average_+3SD)/*Y*_average_−*A*]/*B*^

Here, *Y*_average_ represents the average Raman intensity derived from 10 repeated measurements of the analyte (4-NP or urea) in its powder form. SD is the standard deviation of the Raman signal, calculated from 10 measurements using the formula provided below. *A* and *B* are the intercept and slope, respectively, of the linear equation obtained by plotting the logarithmic SERS intensity (*y*) against the logarithmic concentration (*x*), expressed as (*y* = *A* + *B* × *x*).

SD is calculated *via* the well-known formula:2
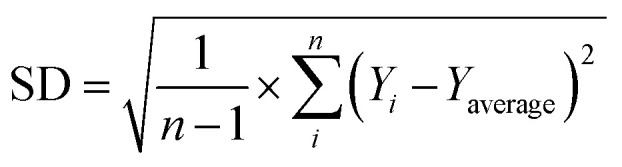
where *n* = 10 is the number of measurements, *Y*_*i*_ is the Raman intensity recorded during the *i*th measurement, and *Y*_average_ is the mean Raman intensity obtained from the 10 repeated measurements of the analyte (4-NP or urea) in pure powdered form.

## 3. Results and discussion

### 3.1. Characterizations of Ag-deposited TiO_2_ nanorod materials

**Fig. 1:**
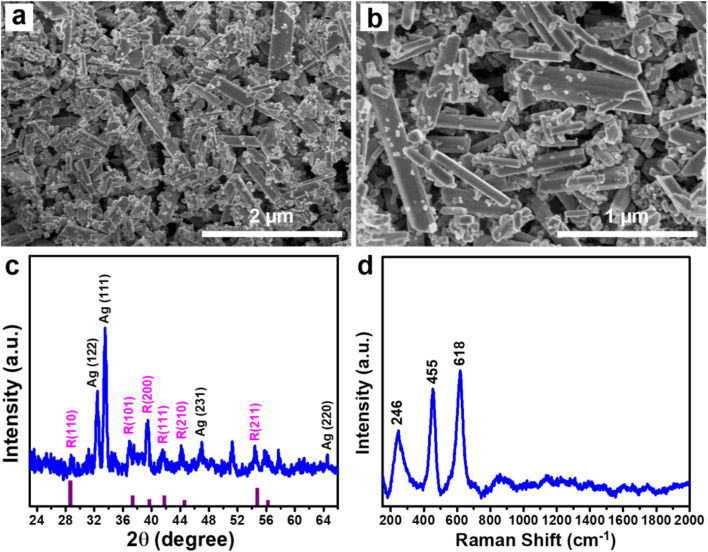
FE-SEM images (a and b); XRD pattern (c) and Raman spectroscopy of Ag/TiO_2_ NR material (d).

The morphology of the Ag/TiO_2_ NR material was investigated using FE-SEM, as shown in Fig. 1a and b. Two distinct shapes were clearly observed: a large rod-like form and smaller spherical particles, which are attributed to the presence of TiO_2_ and Ag. The rod-shaped structures observed in the FE-SEM images are TiO_2_, fabricated using an electrochemical method, as demonstrated in our previous work.^34^ Notably, Ag nanoparticles were uniformly distributed across the TiO_2_ rods, with no observable aggregation or separation from TiO_2_ materials. Fig. 1c presents the XRD pattern, confirming the diffraction peaks of TiO_2_ in the rutile phase at 2*θ* positions of 28.7, 36.8, 39.3, 41.5, 44.1, and 54.3, corresponding to the (110), (101), (200), (111), (210), and (211) crystal planes (JCPDS-PDF card no. 21-1276). Additionally, diffraction peaks at 2*θ* values of 32.4, 33.5, 46.9, and 64.5, characteristic of Ag crystal planes (122), (111), (231), and (220) (JCPDS-PDF card no. 04-0783), confirm the formation and presence of Ag nanoparticles in the Ag/TiO_2_ NR structure. Fig. 1d shows the Raman spectra of the Ag/TiO_2_ NRs, where two characteristic peaks at 455 cm^−1^ and 618 cm^−1^ confirm the presence of rutile TiO_2_ with E_g_ and A_1g_ vibrational modes in its crystal structure.^36^ Moreover, a relatively strong scattering peak at 246 cm^−1^ is observed, which is attributed to the Ag–O vibration, likely due to the interaction between Ag and the TiO_2_ surface.^32^ This suggests a close interaction between Ag and TiO_2_, which is also supported by the FE-SEM images, where Ag nanoparticles are dispersed across the surface of the TiO_2_ nanorods. Furthermore, no additional scattering peaks were detected in the Raman spectrum, indicating that the Ag/TiO_2_ NR material has high purity. Based on these analyses, the Ag/TiO_2_ NR material was successfully synthesized with Ag nanoparticles decorating the surface of rutile TiO_2_ nanorods, demonstrating a close interaction between Ag and TiO_2_.

### 3.2. Application of the PI-PC SERS technique for low Raman cross-section molecules – 4-nitrophenol – a pesticide

4-NP is a commonly used pesticide in agriculture, applied to control a wide range of pests and diseases in crops. As mentioned, 4-NP is a small organic molecule with relatively low polarity compared to highly Raman-active compounds such as rhodamine 6G, which exhibit both strong dipole moments and high Raman cross-sections (∼10^−25^ cm^2^ sr^−1^). In contrast, small molecules like 4-NP typically exhibit Raman cross-sections in the range of 10^−30^ to 10^−27^ cm^2^ sr^−1^, and are thus generally classified as low Raman cross-section analytes.^23,24^ While it is true that 4-NP can exhibit enhanced Raman activity under specific resonance conditions – as demonstrated by Jahncke *et al.*, where only the conjugate base of 4-NP in combination with a 405 nm laser produced a resonance-enhanced Raman signal – such conditions are not commonly applicable in real-world sensing scenarios.^37^ In practical applications, 4-NP is usually present under near-neutral pH conditions, where its conjugate base is not dominant. Moreover, 405 nm laser sources are not commonly used in standard Raman instrumentation due to their high energy, which can cause photodegradation of samples or generate strong fluorescence backgrounds. Therefore, our experiment was designed to closely reflect real-world conditions, utilizing a neutral pH and a low-energy laser source in the near-infrared range (785 nm). Under these conditions, 4-NP remains in a non-resonant state and exhibits a low Raman cross-section. This inherent property can result in a weak scattering probability, leading to low sensing efficiency when using traditional Raman spectroscopy techniques. However, its residue in the environment, particularly in food products, poses significant health risks. Prolonged exposure to 4-NP has been linked to various harmful effects, including potential toxicity to the liver, kidneys, and central nervous system, as well as potential carcinogenic properties.^38^ Beyond its use as a pesticide, 4-NP also serves as an important intermediate in the production of dyes, pharmaceuticals, and petrochemicals. Its discharge into the environment can lead to serious contamination of water and soil, posing substantial risks to both human health and ecological systems. Therefore, monitoring its presence in food, water, and soil is critical to ensure food safety, environmental monitoring and risk prevention. However, the weak SERS signal of 4-nitrophenol presents a challenge, highlighting the need for improved detection sensitivity.

**Fig. 2:**
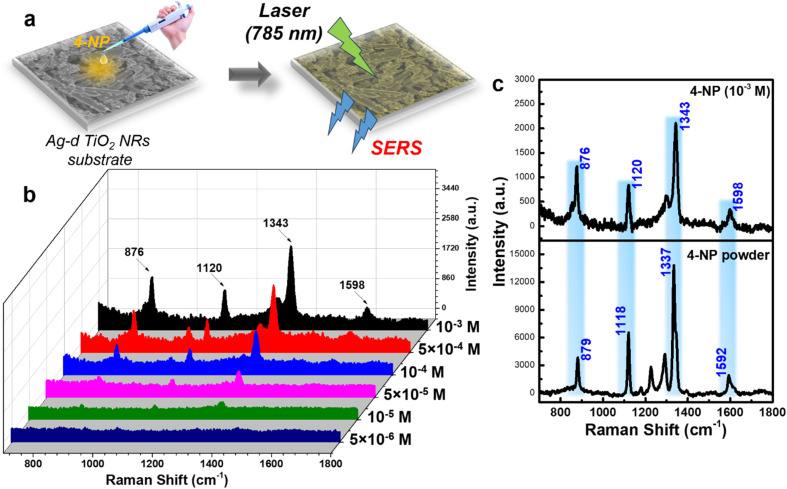
Experiment to collect the 4-NP signal through the normal SERS (without pre-irradiation) from Ag/TiO_2_ NR SERS substrate (a) and SERS spectra of 4-NP in the concentration range of 10^−3^–5 × 10^−6^ M under normal SERS (b). Comparison of the SERS spectrum of 4-NP at a concentration of 10^−3^ M with the Raman spectrum of 4-NP in powdered form (c).

**Fig. 3:**
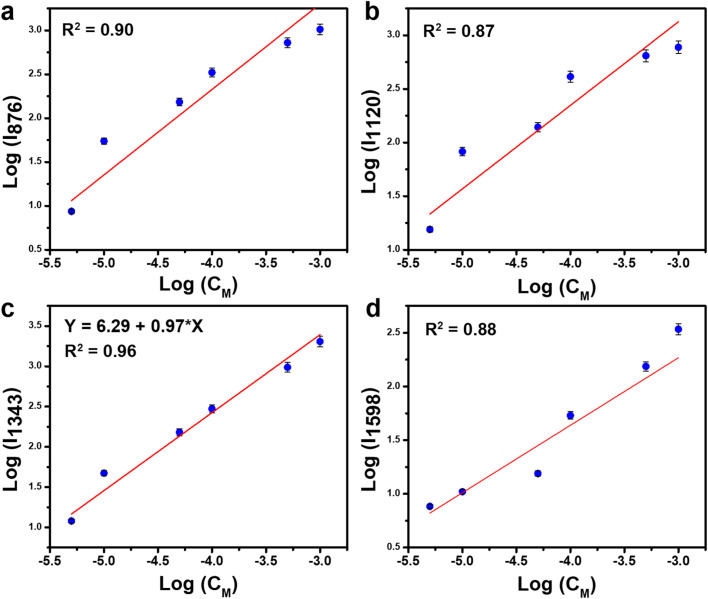
The linear relationship between the concentration of 4-NP and the normal SERS intensity obtained as a logarithmic function at peaks 876 cm^−1^ (a), 1120 cm^−1^ (b), 1343 cm^−1^ (c), and 1598 cm^−1^ (d).

First, the normal SERS experiment was conducted to detect low Raman cross-section molecules for comparing the sensing performance with the PI-PC SERS technique. Fig. 2a briefly illustrates the SERS signal collection from the Ag/TiO_2_ NR SERS substrate in the normal SERS setup. After depositing the Ag/TiO_2_ NRs onto the Al substrate, a 4-NP solution at various concentrations was applied to the substrate surface and allowed to air dry at RT. The SERS signal of 4-NP was then recorded and displayed in Fig. 2b. At a high concentration of 10^−3^ M, distinct scattering peaks at 876, 1120, 1343, and 1598 cm^−1^, characteristic of the 4-NP molecular structure, were observed. First, the observed peaks were compared with the Raman spectrum of pure 4-NP in powdered form (Fig. 2c). A strong match was observed between the characteristic peaks of the pure powder and those in the SERS spectrum of 4-NP at a concentration of 10^−3^ M on the Ag/TiO_2_ NR substrate, confirming the accurate identification of the analyte. These peaks correspond to the bending vibration of the –NO_2_ group, C–H bending vibration, symmetric stretching of the –NO_2_ group, and C=C stretching vibration in the 4-NP molecule.^39,40^ These characteristic peaks gradually decreased as the concentration of 4-NP was reduced. At 10^−5^ M (still considered a high concentration for sensitive platforms like SERS), the characteristic peaks remained visible but with significantly reduced intensity, and completely disappeared at 5 × 10^−6^ M. As expected, the normal SERS technique struggled to detect 4-NP due to its low Raman cross-section. The relationship between 4-NP concentration and the obtained SERS intensity of the characteristic peaks, plotted on a logarithmic scale, is shown in Fig. 3. The 1343 cm^−1^ peak displayed the best linear correlation (*R*^2^ = 0.96), while the peaks at 876, 1120, and 1598 cm^−1^ yielded *R*^2^ values of 0.90, 0.87, and 0.88, respectively. Based on the 1343 cm^−1^ peak, the linear equation relating concentration and intensity was derived as *y* = (6.29 ± 0.15) + (0.97 ± 0.03)*x*, where *x* and *y* represent the logarithms of the 4-NP concentration and SERS intensity, respectively. From this equation, the limit of detection (LOD) was calculated to be 8.1 × 10^−6^ M (detailed LOD determination methods are provided in Section 2.4). With a highly sensitive sensor platform capable of single-molecule detection like SERS, achieving a detection limit of only 10^−6^ M falls short of expectations. This can be improved by applying new techniques and effectively leveraging other effects of the SERS substrate.

**Fig. 4:**
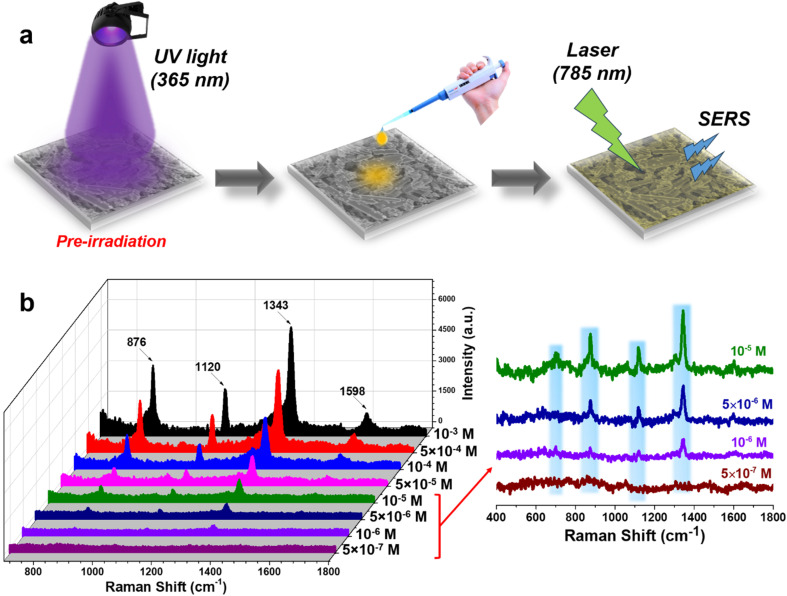
Experiment to collect the 4-NP signal through the PI-PC SERS technique with the pre-irradiation step of the SERS Ag/TiO_2_ NR substrate (a) and the SERS spectra of 4-NP in the concentration range of 10^−3^–5 × 10^−7^ M under the PI-PC SERS method (b).

**Fig. 5:**
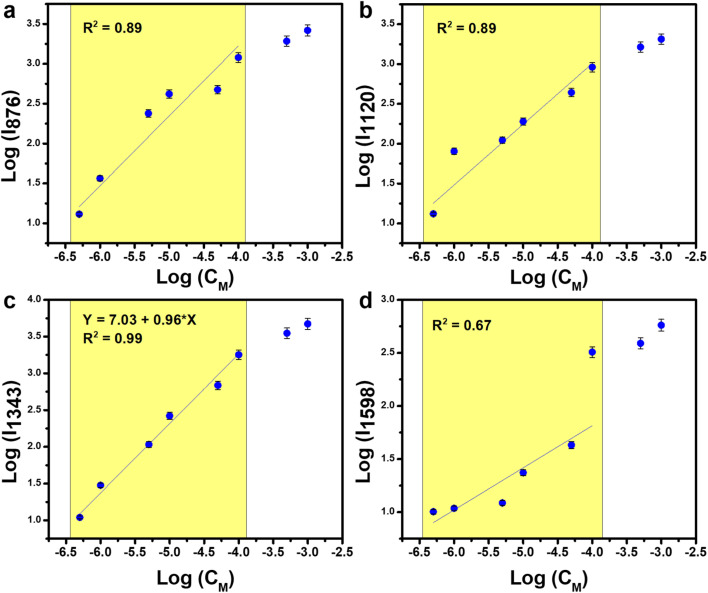
The linear relationship between the concentration of 4-NP and the PI-PC SERS intensity obtained as a logarithmic function at peaks 876 cm^−1^ (a), 1120 cm^−1^ (b), 1343 cm^−1^ (c) and 1598 cm^−1^ (d).

To enhance the detection capability of 4-NP, the PI-PC SERS technique was applied to Ag/TiO_2_ NRs, leveraging the PIERS effect of this nanostructure. Fig. 4a provides a brief overview of the SERS signal collection process for 4-NP using PI-PC SERS, with a pre-irradiation step of the Ag/TiO_2_ NR substrate using UV light (365 nm) for 30 minutes. After irradiation is ceased, solutions of 4-NP at different concentrations are applied to the substrate, and the SERS signals are immediately collected. The resulting sensing data is shown in Fig. 4b. At higher concentrations, 10^−3^ M and 10^−4^ M, the characteristic peaks for 4-NP are clearly visible with high intensity. Notably, at a concentration of 5 × 10^−6^ M, where normal SERS would fail to detect 4-NP, the characteristic peaks are still clearly distinguishable. These peaks remain detectable at a concentration of 10^−6^ M and disappear only at 5 × 10^−7^ M. This demonstrates that the PI-PC SERS technique applied to Ag/TiO_2_ NRs, thanks to the PIERS (PI) effect, significantly enhances the detection capability compared to normal SERS. The linear relationship between 4-NP concentration and the PI-PC SERS signal is quantitatively calculated and displayed in Fig. 5. The peak at 1343 cm^−1^ shows the best linearity, with an *R*^2^ value of 0.99, and a wide linear range from 10^−4^ to 5 × 10^−7^ M. The corresponding linear equation is *y* = (7.03 ± 0.17) + (0.96 ± 0.03)*x*. The limit of detection (LOD) for PI-PC SERS is calculated to be 6.8 × 10^−7^ M, which is 10 times lower than the normal SERS LOD of 8.1 × 10^−6^ M. Table 1 compares the detection performance of 4-NP on various SERS substrates under both conventional SERS and the PI-PC SERS technique. It can be observed that, although highly sophisticated SERS substrates have been developed – such as Au-coated silicon pillars or reduced graphene oxide nanocomposites functionalized with cysteamine and Ag nanoparticles – normal SERS still faces challenges in detecting 4-NP due to its small molecular size and low Raman cross-section, with detection limits typically reaching only around 10^−5^ to 10^−6^ M. Our 2022 study, which applied the PIERS technique on a SERS substrate based on Ag/TiO_2_ (with TiO_2_ in nanoparticle form and anatase phase), achieved significantly enhanced sensing performance compared to normal SERS, reaching a detection limit as low as 1.4 × 10^−6^ M, despite the relatively simple fabrication process of the Ag/TiO_2_ nanoparticles. In this study, by leveraging the PIERS effect through the PI-PC SERS technique on Ag/TiO_2_ NR substrate – with rutile-phase TiO_2_ nanorods – we achieved a further improvement in sensing performance, reaching a detection limit as low as 6.8 × 10^−7^ M. This performance surpasses that of normal SERS using sophisticated substrates and even exceeds the previous PIERS results obtained from Ag/TiO_2_ nanoparticle substrate with anatase-phase TiO_2_ nanoparticles. Therefore, by simply pre-irradiating the Ag/TiO_2_ NR substrate, the detection efficiency for the low Raman cross-section molecule, 4-NP, is enhanced by approximately 10 times and reaches down to the level of 10^−7^ M. This achievement not only improves the detection of low Raman cross-section molecules but also highlights the intriguing enhancement mechanisms of the PI-PC SERS in Ag/TiO_2_ NR substrate.

**Table 1:** Compare the performance of 4-NP detection using the PI-PI SERS technique with that reported in recent studies (PDMS is polydimethylsiloxane, rGOSHAg is a reduced graphene oxide nanocomposite functionalized with cysteamine and Ag nanoparticles)

Substrate	Technique	LOD	Linear range	Reusability	Ref.
Au-coated silicon pillars	SERS	1 × 10^−5^ M	—	—	39
Ag-PDMS	SERS	1 × 10^−6^ M	—	—	41
rGOSHAg	SERS	1 × 10^−6^ M	—	—	39
Ag/TiO_2_ nanoparticles	PIERS	1.4 × 10^−6^ M	1 × 10^−3^–1 × 10^−6^ M	—	32
Ag/TiO_2_ NRs	PI-PC SERS	6.8 × 10^−7^ M	1 × 10^−4^–5 × 10^−7^ M	Yes	This work

**Fig. 6:**
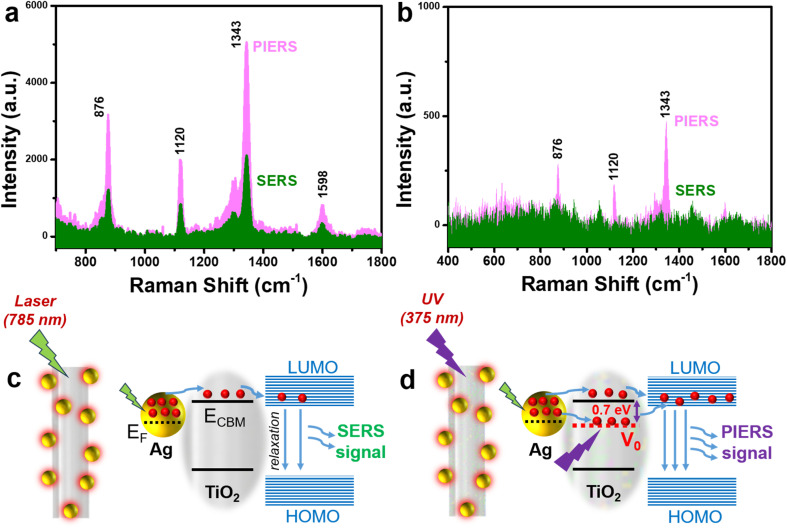
Comparison of the SERS signal intensity obtained for 4-NP in normal SERS and PI-PC SERS at concentrations of 10^−3^ M (a) and 5 × 10^−6^ M (b); signal enhancement mechanism in normal SERS (c) and PI-PC SERS (d).

Fig. 6a and b compare the SERS signals obtained for 4-NP at concentrations of 10^−3^ M and 5 × 10^−6^ M in both normal SERS and PI-PC SERS. Based on the signal intensity of 4-NP at the 1343 cm^−1^ peak – selected for its strong enhancement and its association with the characteristic –NO_2_ group of 4-NP – the PI-PC SERS technique demonstrated signal enhancements of approximately 2.4-fold and 8.9-fold at concentrations of 10^−3^ M and 5 × 10^−6^ M, respectively, compared to normal SERS. The SERS signal enhancement driven by the PIERS effect has also been demonstrated in other nanostructures and analytes. Ben-Jaber *et al.* observed significant PIERS-induced enhancement using Au/TiO_2_ nanoparticle substrates across a wide range of molecular targets, including organic dyes, biomolecules, and explosives.^16^ Similarly, Barbillon *et al.* reported up to a 7.52-fold enhancement for thiophenol compared to conventional SERS using Au/ZnO nanoparticle structures.^42^ The enhanced SERS signal in PI-PC SERS, attributed to the PIERS effect, arises from photoelectric interaction and the energy level transitions between Ag and TiO_2_ in the Ag/TiO_2_ NR structure, where the presence of TiO_2_ facilitates more efficient charge transfer between Ag and 4-NP.^29,30^ During the measurement, the localized surface plasmon resonance (LSPR) of the Ag nanoparticles excites free charges that oscillate around the nanoparticle surfaces.^43^ These excited charges can transfer to nearby analyte molecules, and the recovery of these charges within the analyte molecules generates characteristic Raman signals corresponding to molecular vibrations.^44^ The transferred charge from Ag to the analyte tends to reach the lowest unoccupied molecular orbital (LUMO) energy level (in this case, for the 4-NP molecule), where the charges then relax to the highest occupied molecular orbital (HOMO) of 4-NP, contributing to the overall SERS signal (Fig. 6c).^45,46^ The TiO_2_ component in the SERS substrate enhances the charge transfer efficiency between Ag and 4-NP. Upon applying the PI-PC SERS technique to the Ag/TiO_2_ NR structure by pre-irradiating the substrate with UV light at a wavelength of 365 nm (to activate PIERS effect), this illumination causes oxygen atoms on the TiO_2_ surface to be ejected, forming oxygen vacancy sites – a phenomenon previously documented in other studies.^16,30^ Mezhenny *et al.* demonstrated that UV irradiation induces the formation of oxygen vacancies on the surface of TiO_2_, which can remain stable for up to 48 hours after the irradiation ceases.^47^ These oxygen vacancies create a temporary intermediate energy level between the valence band and the conduction band of TiO_2_, providing a site where electrons can be accepted.^48^ This temporary energy level was demonstrated by Henrich *et al.* to lie approximately 0.7 eV below the conduction band of TiO_2_.^48^ This intermediate energy level in TiO_2_ facilitates more efficient charge transfer from the LSPR of Ag to TiO_2_, thus increasing the probability and number of charges transferred to the 4-NP molecule.^49,50^ As a result, the SERS signal is significantly enhanced in the PI-PC SERS technique compared to normal SERS (Fig. 6d).

**Fig. 7:**
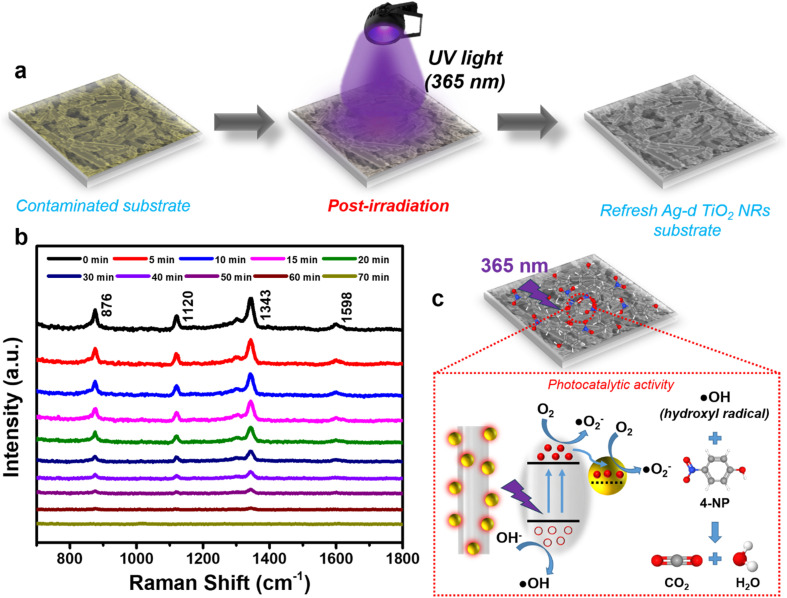
Post-irradiation experiment to activate the photocatalytic activity of the Ag/TiO_2_ NR SERS substrate for the removal of the contaminant 4-NP after the sensing process (a); SERS spectra monitoring the photocatalytic degradation of 4-NP on the Ag/TiO_2_ NR substrate (b) and the photocatalytic mechanism of 4-NP degradation by the Ag/TiO_2_ NR substrate (c).

The optoelectronic phenomenon of the Ag/TiO_2_ NR structure not only proves highly effective in enhancing the detection of 4-NP through the PIERS effect but also offers the potential for self-cleaning and reuse of the SERS substrate. The Ag/TiO_2_ structure is widely known for its superior photocatalytic efficiency.^51,52^ Leveraging this property, the Ag/TiO_2_ NR SERS substrate, which contains residual 4-NP molecules after the sensing process, can be treated to self-clean its surface and be reused for subsequent analyses. This approach can reduce both the effort and cost involved in fabricating new SERS substrates while also minimizing waste generated from discarded substrates after each analysis. After the sensing process, the contaminated Ag/TiO_2_ NR substrate, which has 4-NP molecules on its surface, was dropped with a sufficient of water and then subjected to UV irradiation at a wavelength of 365 nm to activate the photocatalytic decomposition property of the Ag/TiO_2_ structure (Fig. 7a). The effectiveness of removing residual 4-NP molecules is monitored through the SERS signal obtained from the Ag/TiO_2_ substrate. The results are depicted in Fig. 7b. At time point 0 minutes, the SERS signal of 4-NP at a concentration of 5 × 10^−4^ M is measured before the photocatalytic process begins. Subsequently, time intervals of 5, 10, 20, 30… minutes of the degradation reaction are monitored using SERS spectra to assess the reduction in 4-NP concentration on the contaminated SERS substrate. As time progresses, the intensity of the characteristic peaks of 4-NP diminishes, indicating the gradual breakdown of molecular bonds within the 4-NP structure. This demonstrates that the photocatalytic activity of the Ag/TiO_2_ NR substrate is effectively decomposing the 4-NP molecules present on its surface. After 70 minutes of irradiation and reaction, the SERS spectrum of the contaminated Ag/TiO_2_ NR substrate no longer shows characteristic peaks of 4-NP, implying that the residual 4-NP molecules on the surface of the SERS substrate have been sufficiently decomposed, and their signal no longer influences the overall SERS response.

Fig. 7c succinctly outlines the mechanism behind the photocatalytic decomposition of 4-NP by the Ag/TiO_2_ NR substrate. Upon UV irradiation at a wavelength of 365 nm, the electrons in the valence band of the TiO_2_ nanorods with the rutile crystal phase are excited, jumping to the conduction band and creating electron–hole pairs.^53^ These electron–hole pairs then react with water and oxygen in the environment to produce hydroxyl radicals (·OH), which directly interact with the 4-NP molecules, leading to the formation of CO_2_, water, and other by-products.^53,54^ The presence of Ag nanoparticles further enhances the photocatalytic decomposition activity of the Ag/TiO_2_ NR structure by reducing the recombination rate of the electron–hole pairs on TiO_2_, thereby increasing the number of electron–hole pairs available to participate in the reaction.^51,52^ Ultimately, after the self-cleaning process, the Ag/TiO_2_ NR SERS substrate is heated at 60 °C to remove water and by-products, resulting in a renewable and reusable SERS substrate.

**Fig. 8:**
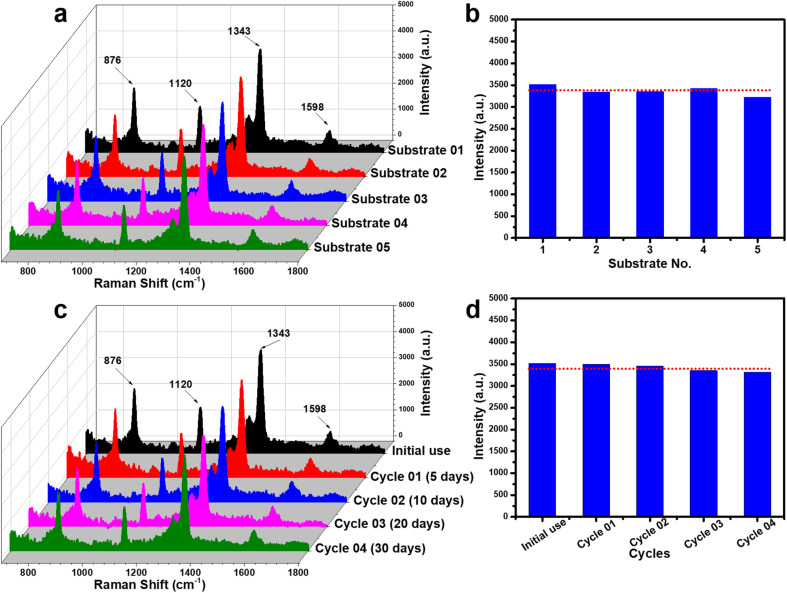
Evaluation of the reproducibility (a and b) and reusability (c and d) of the PI-PC SERS technique for detecting 4-NP at a concentration of 5 × 10^−4^ M. (a) SERS spectra of 4-NP collected from five different Ag/TiO_2_ NR substrates prepared in separate batches. (b) Comparison of the 1343 cm^−1^ peak intensity obtained from the five different substrates. (c) SERS spectra of 4-NP collected from a substrate over multiple reuse cycles at different storage time points. (d) Comparison of the 1343 cm^−1^ peak intensity across the reuse cycles.

To assess the reliability of the PI-PC SERS technique using Ag/TiO_2_ NR substrates, two key parameters were examined: reproducibility and reusability. Reproducibility was evaluated by collecting SERS signals of 4-NP (5 × 10^−4^ M) from five independently prepared Ag/TiO_2_ NR substrates, each subjected to the same PI-PC SERS protocols. Reusability was assessed by reapplying one substrate across multiple cycles at various storage intervals. The results are presented in Fig. 8. For reproducibility, five independent experiments were conducted, encompassing substrate fabrication, pre-irradiation, and signal collection. As shown in Fig. 8a, the SERS spectra from all five substrates display consistent characteristic peaks of 4-NP. The intensity of the 1343 cm^−1^ peak – selected for its strong linearity – was used to calculate the relative standard deviation (RSD), which was 8.22% (Fig. 8b). This low RSD value indicates excellent reproducibility of the PI-PC SERS technique with Ag/TiO_2_ NR substrate. The reusability of the substrate, enabled by its photocatalytic self-cleaning property, was also investigated. After each use, the substrate was cleaned and reused to detect 4-NP. Fig. 8c shows that the characteristic SERS peaks of 4-NP remain clearly visible across reuse cycles, with negligible intensity variation. As depicted in Fig. 8d, while the 1343 cm^−1^ peak shows a slight decline in intensity with successive uses, the signal remains strong, demonstrating robust reusability. Additionally, the long-term stability of the substrate was examined by storing the Ag/TiO_2_ NRs in sealed containers protected from light and conducting reuse tests after 5, 10, 20, and 30 days. The SERS signals obtained at these intervals (Fig. 8c and d) remained stable, confirming the substrate's good stability. From these results, the Ag/TiO_2_ NR substrate, when coupled with the PI-PC SERS strategy, exhibits good reproducibility, reusability, and storage stability, highlighting its strong reliability.

### 3.3. Application of the PI-PC SERS technique for low Raman cross-section molecules – urea – a biomarker

Urea, a diamide derivative of carbonic acid, is a vital nitrogen-containing compound and a crucial biomarker present in human blood and urine. It plays a critical role in clinical diagnoses related to kidney, liver, and heart diseases.^55,56^ Beyond clinical applications, urea detection is important in agriculture, environmental monitoring, and food safety. Elevated urea levels in soil and water can indicate nutrient imbalances or contamination from agricultural runoff, which affects crop health and water quality.^57,58^ In food safety, urea contamination in food products can signal improper handling or excessive fertilizer use, posing potential health risks.^59^ Despite its significance, urea has a low Raman scattering cross-section, which limits the effectiveness of the SERS technique for detecting this biomarker due to weak SERS signals. Enhancing the detection sensitivity for urea could offer valuable applications in early diagnosis, healthcare, food safety and environmental protection. The PI-PC SERS technique, applied to Ag/TiO_2_ NR SERS substrates, was further explored to improve the detection of this important molecule. The Ag/TiO_2_ NR SERS substrates, cleaned after the 4-NP experiments, were reused in this urea experiment to assess their reusability.

**Fig. 9:**
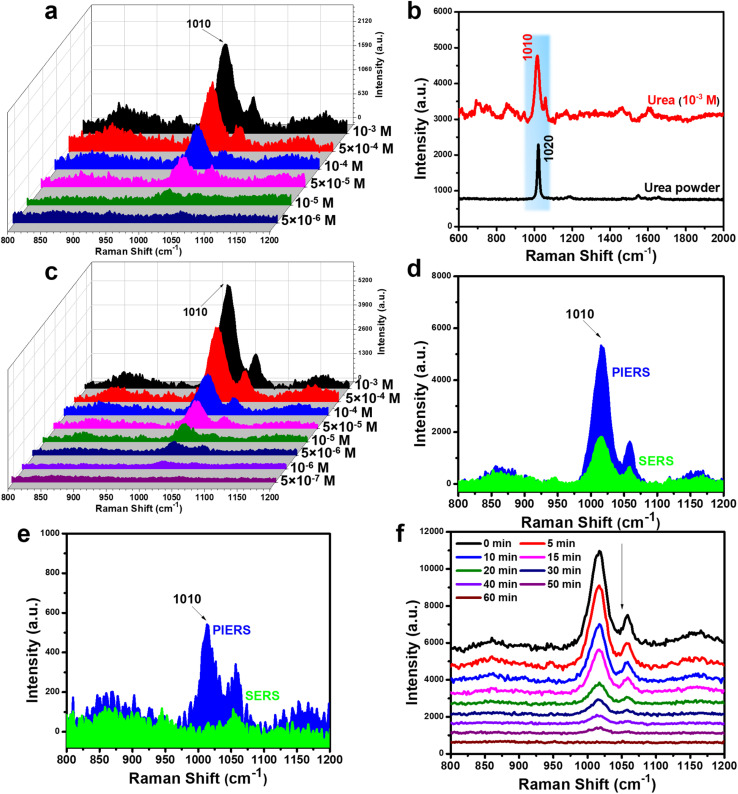
SERS spectra of urea in the concentration range of 10^−3^–5 × 10^−6^ M under normal SERS (a); comparison of the SERS spectrum of urea at a concentration of 10^−3^ M with the Raman spectrum of urea in powder form (b). SERS spectra of urea in the concentration range of 10^−3^–5 × 10^−7^ M under PI-PC SERS technique (c); comparison of the SERS signal intensity of urea obtained in normal SERS and PI-PC SERS at concentrations of 10^−3^ M (d) and 5 × 10^−7^ M (e); SERS spectra monitoring the photocatalytic degradation of urea on Ag/TiO_2_ NR substrate (f).

Fig. 9a shows the results obtained from normal SERS experiments for urea. As a small organic molecule consisting of a carbonyl group (C=O) and two amino groups (–NH_2_), urea adsorbed on SERS substrates produced a straightforward SERS spectrum, characterized by a distinct peak at 1010 cm^−1^ corresponding to the C–N stretching vibration.^60^ Compared to the Raman spectrum of pure urea in powdered state, the SERS spectrum obtained from the urea solution shows a slight peak shift, with the powder spectrum displaying a characteristic peak at 1020 cm^−1^ (Fig. 9b). This characteristic peak appeared clearly with relatively high intensity at a concentration of 10^−3^ M and gradually decreased at lower concentrations. At a concentration of 10^−5^ M (Fig. 9a), the characteristic peak was still observable but very weak, and it completely disappeared at a concentration of 5 × 10^−6^ M. Additionally, the SERS spectrum of urea used on the reused substrate from the 4-NP experiment showed no unusual scattering peaks and did not affect the ability to detect urea. This demonstrates the good reusability of this SERS substrate through the PI-PC SERS technique. The calculated LOD value in this normal SERS case was 8.9 × 10^−6^ M. Fig. 9c shows the detection results for urea when applying the PI-PC SERS technique on the Ag/TiO_2_ NR SERS substrate over the concentration range of 10^−3^–5 × 10^−7^ M. When comparing the SERS signal intensity at a concentration of 10^−3^ M, PI-PC SERS clearly shows the superior detection performance compared to normal SERS (Fig. 9d). The 1010 cm^−1^ peak in the PI-PC SERS case appears more clearly and with significantly higher intensity than in the normal SERS. At a concentration of 5 × 10^−6^ M, normal SERS could not detect urea, while the characteristic peak of urea remained clearly visible when applying PI-PC SERS (Fig. 9e). The LOD value in the PI-PC SERS was 6.9 × 10^−7^ M, enhanced by over 10 times compared to normal SERS. Table 2 presents a comparison of urea sensing performance across various SERS substrates using both normal SERS and the PI-PC SERS technique. Although these substrates – such as Ag dendritic structures, highly ordered Ag/Cu nanostructure arrays, and Au@Ag nanoparticles – were meticulously engineered, their detection capability for urea, a small molecule with a low Raman scattering cross-section, remains limited to the range of 10^−3^–10^−4^ M under normal SERS. By employing the PIERS technique on Ag/TiO_2_ substrates containing anatase-phase TiO_2_ nanoparticles, the detection limit is significantly enhanced, reaching 10^−6^ M. Remarkably, the Ag/TiO_2_ NRs used in this study, featuring rutile-phase TiO_2_ in rod morphology, enable an even lower detection limit of 10^−7^ M when using the PI-PC SERS technique. It should be noted that PI-PC SERS synergistically integrates PIERS and photocatalysis, with the observed signal enhancement attributed to the PIERS effect. This substantial improvement in urea detection underscores the excellent SERS enhancement capability of the Ag/TiO_2_ NR substrate when applying the PI-PC SERS approach. The Ag/TiO_2_ NR SERS substrate contaminated with urea was also cleaned through the photocatalytic effect (Fig. 9f), and after 60 minutes of post-irradiation, the urea signal was completely removed.

**Table 2:** Compare the performance of urea detection using the PI-PI SERS technique with that reported in recent studies

Substrate	Technique	LOD	Linear range	Reusability	Ref.
Ag dendrites	SERS	0.2 mg mL^−1^ (∼3.33 × 10^−3^ M)	0.17–3.33 × 10^−3^ M	—	61
Au/Cu nanostructure arrays	SERS	1 × 10^−3^ M	0.3–1 × 10^−3^ M	—	19
Ag–Au compound	SERS	1 × 10^−3^ M	2 × 10^−2^–1 × 10^−3^ M	—	62
Au@Ag NPs	SERS	5 mg dL^−1^ (∼8.33 × 10^−4^ M)	1.3 × 10^−3^–8.3 × 10^−4^ M	—	63
Ag/TiO_2_ nanoparticles	PIERS	4.6 × 10^−6^ M	1 × 10^−3^–1 × 10^−6^ M	—	32
Ag/TiO_2_ NRs	PI-PC SERS	6.9 × 10^−7^ M	1 × 10^−4^–5 × 10^−7^ M	Yes	This work

**Fig. 10:**
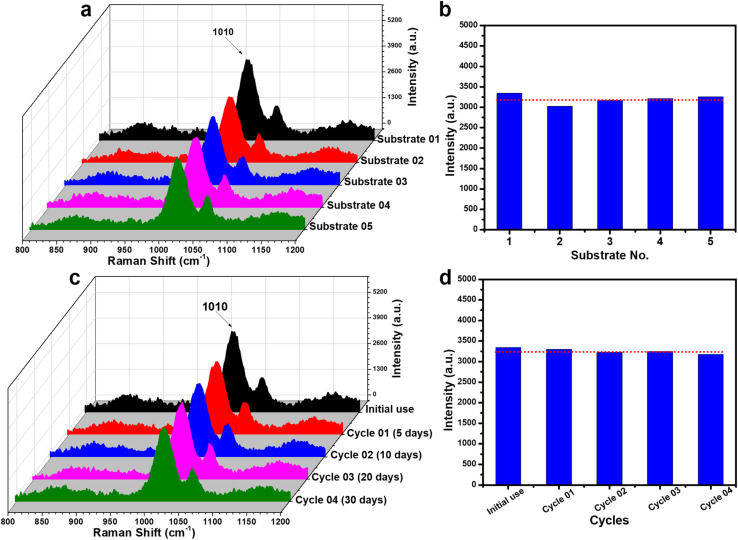
Evaluation of the reproducibility (a and b) and reusability (c and d) of the PI-PC SERS technique for detecting urea at a concentration of 5 × 10^−4^ M. (a) SERS spectra of urea collected from five different Ag/TiO_2_ NR substrates prepared in separate batches. (b) Comparison of the 1010 cm^−1^ peak intensity obtained from the five different substrates. (c) SERS spectra of urea collected from a substrate over multiple reuse cycles at different storage time points. (d) Comparison of the 1010 cm^−1^ peak intensity across the reuse cycles.

Similar to the case of 4-NP, the reliability of the PI-PC SERS technique using Ag/TiO_2_ NR substrates was also further evaluated for urea at a concentration of 5 × 10^−4^ M by assessing reproducibility and reusability. The experimental procedure was conducted in the same manner as with 4-NP to ensure repeatability. The results shown in Fig. 10a and b confirm the high reproducibility of the PI-PC SERS technique for urea, with a RSD value of 6.34%. Furthermore, Fig. 10c and d demonstrate the excellent reusability and stability of the Ag/TiO_2_ NR substrate across multiple reuse cycles and various storage time points. These results, together with the findings obtained for 4-NP, further highlight the high reliability of combining Ag/TiO_2_ NR substrates with the PI-PC SERS technique, which not only offers sensitive sensing performance, good reproducibility, reusability, and stability, but also demonstrates applicability to various low Raman cross-section molecules.

## 4. Conclusions

This study successfully demonstrates the application of the photo-induced photo-catalytic (PI-PC SERS) technique using Ag-deposited TiO_2_ nanorod-based SERS substrates to enhance the detection of low Raman cross-section molecules, specifically the pesticide 4-NP and the biomarker urea. Normal SERS struggles with detecting such molecules, with detection limits restricted to 10^−6^ M. By leveraging the unique PIERS effect of Ag/TiO_2_ NRs, PI-PC SERS significantly improves detection sensitivity, achieving a tenfold enhancement and lowering the detection limit to 10^−7^ M. Beyond its enhanced detection capability, PI-PC SERS harnesses the high photocatalytic activity of Ag/TiO_2_ NRs to degrade residual analyte molecules post-sensing, enabling an efficient self-cleaning and reusable SERS platform. These advancements not only improve detection efficiency but also enhance the cost-effectiveness and sustainability of SERS-based analytical techniques. The findings of this study highlight the transformative potential of PI-PC SERS in overcoming the challenge of detecting low Raman cross-section molecules, paving the way for future research in ultrasensitive sensing while expanding its applications in food safety, biomedical diagnostics, and many related areas. However, some limitations remain. This study tested on two model low Raman cross-section analytes (4-NP and urea); further investigations are needed to confirm PI-PC SERS performance across a broader range of such molecules. Additionally, its practical applicability should be further validated using complex real-world samples to ensure reliability and reproducibility under practical conditions.

## Data availability

All experimental data, including the characterization of the Ag-deposited TiO_2_ nanorod substrate and the detection results for 4-nitrophenol and urea molecules, are included in the manuscript.

## Author contributions

Q. D. Mai: conceptualization, methodology, investigation, formal analysis, data curation, supervision, writing – original draft; D. T. H. Trang: formal analysis, investigation, validation; N. T. Loan: validation, investigation; N. H. T. Thi: validation, formal analysis; O. V. Hoang: validation, investigation; T. N. Bach: validation, investigation; N. Q. Hoa: validation, investigation; A. T. Pham: methodology, supervision; A. T. Le: conceptualization, methodology, supervision, project administration, writing – review & editing.

## Conflicts of interest

The authors confirm that no financial interests or personal relationships exist that could have influenced the findings presented in this paper.
